# Risk and resilience-based restoration optimization of transportation infrastructures under uncertainty

**DOI:** 10.1371/journal.pone.0308138

**Published:** 2024-08-01

**Authors:** Juanjuan Lin, Qizhou Hu, Wangbing Lin, Minjia Tan

**Affiliations:** 1 School of Automation, Nanjing University of Science and Technology, Nanjing, China; 2 School of Urban Construction and Design, Taizhou Institute of Science and Technology, Nanjing University of Science and Technology, Taizhou, China; 3 School of Ocean College, Jiangsu University of Science and Technology, Zhenjiang, China; Aalto University, FINLAND

## Abstract

Disruptive events cause decreased functionality of transportation infrastructures and enormous financial losses. An effective way to reduce the effects of negative consequences is to establish an optimal restoration plan, which is recognized as a method for resilience enhancement and risk reduction in the transportation system. This study takes the total travel time as the resilience measure to formulate a bilevel optimization model for a given scenario. However, the uncertainties involved in restoration activities cannot be overlooked. In this context, the inherent uncertainty is represented with a set of scenarios generated via the Latin hypercube technique. To assess the risk under uncertainty, a conditional value at risk with regret (CVaR-R) measure is introduced when considering the existence of worst-case scenarios. Then, the bilevel programming model is transformed from the deterministic case to the stochastic case, where the upper-level problem determines the restoration sequence to minimize CVaR-R and the lower-level problem is a traffic assignment problem. An integrated framework based on a novel genetic algorithm and the Frank—Wolfe algorithm is designed to solve the stochastic model. Numerical experiments are conducted to demonstrate the properties of the proposed bilevel programming model and the performance of the solution algorithm. The proposed methodology provides new insights into the restoration optimization problem, which provides a reference for emergency decision-making.

## 1 Introduction

Transportation infrastructures are important components of modern cities and play a vital role in promoting social stability and economic growth. Unfortunately, they are susceptible to disruptive events, which induce enormous financial losses and physical damage. For example, the 2008 Sichuan earthquake caused significant physical damage to 21 highways and destroyed 11 provincial roads, leading to great economic losses [1]. The Tohoku earthquake and tsunami in 2011 destroyed approximately 1,500 highway bridges and numerous rail bridges, with an estimated $170 billion in economic damage [2, 3]. In most cases, preventing disruptive events from occurring and controlling losses during events are difficult to achieve. Furthermore, the extensive restoration of disrupted transportation infrastructures usually requires a considerable amount of time and cost to return them to their original state, causing the transportation system to remain partially disrupted during the rehabilitation phase. Therefore, it is vital to develop an efficient restoration plan with limited resources that can be used to restore disrupted transportation infrastructures as quickly as possible.

Resilience is particularly crucial because of the tremendous ripple effects of disruptive events on infrastructures. For this reason, it has become a key indicator for evaluating the performance of transportation networks in recent years and thus has increasingly attracted attention. Transportation infrastructure resilience can be summarized as the ability to reduce the magnitude and/or duration of disruptive events [4]. Many studies have been conducted to optimize the postdisaster restoration of transportation networks via the quantification of system resilience. Depending on whether or not the effect of varying traffic flow volume is taken into consideration, the resilience measures can be divided into two main categories: topology-based and system-based [5, 6]. The former includes a range of graph theory-based measures such as connectivity [7], accessibility [8], and betweenness centrality [9], while the latter encompasses resilience measures that consider the effects of congestion, such as travel time [10–12], travel cost [13, 14], and travel demand [15]. Among various existing resilience measures, travel time is one of the most widely adopted [16]. For instance, Karamlou and Bocchini [10] considered the total travel time and total travel distance to determine the optimal bridge restoration sequence in their resilience analysis. Kaviani et al. [11] aimed to improve the resilience of regional road networks by maximizing the total travel time and demand satisfaction. Both Yoon et al. [17] and Gokalp et al. [18] applied the total travel time to restore damaged components when only one infrastructure could be repaired at a time.

For transportation networks where disruptive events occur, some scholars have coupled the risk and resilience of the networks in their studies. Zhao [19] formulated a resilience recovery strategy to reduce the losses of the road network caused by risk, in which the users’ choice behavior and traffic flow were not included in the analysis. Sohouenou and Neves [20] focused on comparing link repair sequences in terms of resilience to reduce risk consequences but failed to consider the effect of the duration of repair operations performed on individual links. Caliendo et al. [21] established a traffic simulation model for evaluating the resilience of a twin-tube road tunnel and the risk level of tunnel users. However, their model suffers from critical limitations: it measures risk in terms of fatalities and fails to consider the interactions between tunnel users.

Nevertheless, the above studies suffer a common shortcoming that they are all based on deterministic assumptions in which all information on disrupted infrastructures can be obtained. Whereas, in reality, it is difficult to obtain accurate information in a timely manner because of the suddenness of disruptive events and the limited ability to gather information. Hence, some researchers have investigated the uncertainty of the restoration issue in the field of transportation. The highlights of the literature related to the uncertainty of the restoration scheduling problem are listed in chronological order in Table 1, which focuses on four aspects, namely, types of resilience measure, types of uncertainty, the traffic assignment models/principles used during the restoration process and types of risk measure.

**Table 1:** Literature on transportation network restoration.

Literature	Resilience measures	Uncertainty	Traffic assignment model/ principles used	Risk measures
Aydin et al. [22]	Connectivity	Recovery time	Shortest path	
Zhang et al. [23]	Total recovery time;	Recovery time	Shortest path	
Li et al. [24]	Recovery rapidity; cumulative loss	Recovery time	UE	
Hosseini et al. [25]	Resilience loss	Recovery time	Shortest path	
Zhang et al. [26]	Ratio of system recovery	Disrupted capacity; recovery speed	Simulation	
Zhang et al. [27]	Average travel time	Damage index; delay duration; recovery speed	UE	
This study	Total travel time	Recovery time	UE	CVaR-R

As shown in Table 1, Aydin et al. [22] presented a framework to evaluate recovery strategies of rural transportation networks following earthquakes and earthquake-triggered landslides, considering the uncertainties associated with recovery times. However, the proposed framework has two limitations: one is that it does not consider the cost of allocating resources, and the other is that the road recovery sequence is based on proximity. Zhang et al. [23], Li et al. [24] and Hosseini et al. [25] also modeled the uncertainty of restoration durations in their resilience analysis. Zhang et al. [23] developed a novel methodology for optimizing the restoration schedules of road-bridge transportation networks, which measures network performance via a connectivity reliability-based metric and thus fails to reflect the characteristics of network traffic flow during the restoration phase. Assuming that the restoration duration of each disrupted road segment follows a normal distribution, Li et al. [24] proposed a stochastic programming model for the restoration of the freight network to maximize system resilience. Nevertheless, they quantified network performance on the basis of connectivity rather than system-based measures. Hosseini et al. [25] presented an integrated framework to optimize the restoration sequences following earthquakes, where the resilience measure used is a topology-based index and the effects of traffic congestion are ignored. In addition, Zhang et al. [26] developed a component restoration function to quantify resilience, which takes into account uncertain residual capacity and recovery speed. Here, they combined a probabilistic solution discovery algorithm with a stochastic ranking approach to develop a restoration plan, which is clearly inappropriate for large-scale transportation networks. More recently, Zhang et al. [27] developed a resilience-based methodology to determine the optimal restoration sequences for bridge networks, taking into account the uncertainties regarding the bridge damage level, delay duration and recovery speed. In their work, network performance is measured by average travel time, with the limitation that the methodology might ignore the existence of Braess paradox links during the recovery process. Overall, the uncertainty regarding the recovery time of disrupted components is the most widely considered among these existing studies.

Notably, most of the above studies did not consider travelers’ route choice behavior. Disruptive events decrease the infrastructure capacity, and the capacity of the repaired infrastructure returns to its designed level, which would cause travelers to adjust their route choices according to the changes in infrastructure capacity within each subperiod. Travelers usually choose their route that satisfies their utility maximization, i.e., the travel time spent on their chosen route is no more than that of any other unchosen route. Thus, the user equilibrium (UE) traffic assignment approach is more suitable for analyzing the properties of the restoration scheduling problem.

Moreover, as mentioned in Table 1, previous studies only introduced uncertainty into the transportation network restoration scheduling problem, to establish a stochastic model. These stochastic models usually seek to maximize system resilience while ignoring the risks associated with uncertainty once the resulting optimal or risk-neutral restoration plan is implemented [28]. Thus, when using a stochastic model, it is crucial to consider the risk management associated with the restoration plan to identify the possible worst-case scenarios and change the plan accordingly [29]. Risk management strategies have been considered in electric power systems [28, 29] and water systems [30]. However, to our knowledge, they have not been examined in transportation systems.

To address the abovementioned challenges, this study proposes a risk- and resilience-based optimization approach for the restoration scheduling problem with stochastic recovery time. To describe the uncertainty, a set of recovery time scenarios is generated according to the Latin hypercube sampling approach. Moreover, the network resilience is measured by the total travel time spent over the entire restoration process, while the system risk is measured via the conditional value at risk with regret (CVaR-R). To obtain the optimal CVaR-R value and determine the restoration plan under stochastic recovery time, a bilevel programming model is proposed. The upper-level problem is to determine the optimal restoration sequence considering CVaR-R minimization, while the lower-level problem is the traffic assignment model that captures travelers’ responses to changes in transportation network capacity.

To sum up, the main contributions of the present study are summarized as:

1. A risk measure, namely, CVaR-R, is proposed to manage the risk that the system resilience for worst-case scenarios is lower than expected, which, to the best of the authors’ knowledge, is the first effort in the literature to integrate the risk measure with the resilience measure for transportation network restoration under uncertainty.
2. A bilevel stochastic optimization model transformed from the deterministic case is developed to determine the optimal restoration plan based on the proposed risk and resilience measure, while travelers’ responses to changes in the transportation network topology and capacity are captured via a lower-level traffic assignment model.
3. A novel genetic algorithm (GA) is employed to determine the ideal solution for each recovery time scenario and to find the best solution for this set of scenarios under stochastic recovery time. The effectiveness of the proposed methodology is demonstrated via sensitivity analysis, which provides support to decision-makers responsible for formulating emergency-response strategies for transportation systems.

The remainder of this study is organized as follows. Section 2 introduces the mathematical formulations and the solution approach used in this study. Section 3 presents numerical examples to illustrate the advantages of the suggested methodology and the influence of sensitive factors on the solution, followed by a discussion. Finally, concluding remarks and future extensions are provided in section 4.

## 2 Methodology

### 2.1 Problem description

The study problem can be described as follows: Given a transportation network *G* = (*N*, *A*), there is a set of nodes *N* (including origins, destinations and road intersections) and a set of directed links *A* (representing road segments). Obviously, the occurrence of disruptive events could lead to damage to transportation infrastructures (i.e., nodes and links), along with decreasing the capacity of the infrastructures, resulting in poor network performance and low efficiency for travelers.

A unique goal of this work is to maximize the system resilience by minimizing the total travel time for a given disruptive event scenario. In this study, the restoration phase is split into time periods. Travelers adjust their route choices based on the changing capacity of the infrastructures during the restoration process, which is described by a traffic assignment model within each period.

Another main task is to focus on uncertainties to decrease losses in the worst-case scenarios. Considering the limited ability of people to gather information, the restoration process is complicated and stochastic; thus, some parameters, such as the recovery time for each disrupted infrastructure and the required restoration resources, introduce uncertainty into the restoration environment. Therefore, the Latin hypercube technique is employed to generate a set of scenarios that account for stochastic recovery time, and the system risk under uncertainty is minimized via CVaR-R.

**Fig 1:**
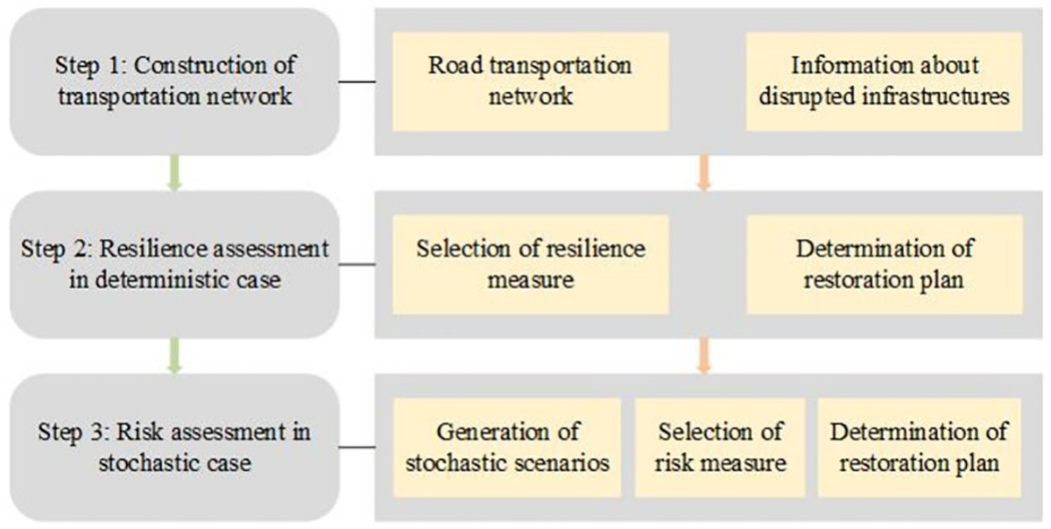
The workflow in this study.

Accordingly, to restore the transportation infrastructure to its original state, resilience and risk measures are employed to determine the optimal restoration strategy during the restoration process under limited resources. Fig 1 presents the workflow used in this study, which is derived from the above research objectives and ideas. Step 1 is relatively simple. The system assessment in Steps 2 and 3 is introduced in the following subsections.

To facilitate the discussion of the restoration scheduling problem, the following assumptions are made.

A1. The disrupted infrastructures include only the links that connect nodes because node degradation can be regarded as link degradation.
A2. It is assumed that different resources can be converted into one monetary value. Thus, the resource constraints can be expressed as a uniform budget constraint associated with each period.
A3. The resources required for each disrupted link are given and deterministic because this paper addresses the stochasticity for the restoration period only.
A4. The restoration of each disrupted link exists along a continuum. Once the restoration of a disrupted link is started, the resources available in each period prioritize the need of the link rather than other disrupted links.
A5. The capacity of the disrupted link remains in the initial damaged state and immediately returns to its designed level as soon as that link is fully repaired.
A6. The travel demand between each OD pair is assumed to be stationary over the course of recovery.

### 2.2 Mathematical modeling

#### 2.2.1 Deterministic bilevel programming

In this subsection, we provide a resilience-based bilevel formulation to determine the optimal restoration plan for a given scenario, that is, the deterministic case.

*(1) Upper-level formulation*.

In the case of certainty, the recovery time vector **T**_*l*_ = (*T*_*a*_,∀*a* ∈ *R*) for scenario *l* is fixed and given. By applying the total travel time as the resilience measure, the upper-level problem focuses especially on maximizing system resilience throughout the whole restoration horizon. The objective function of the upper-level problem minimizes the total travel time defined as the sum of the travel time spent by each traveler in the transportation network, which can be expressed as:

$$\underset{s,v,y,\eta }{\text{min}}Z\left({\text{T}}_{l}\right)=\sum_{\tau \in H}\sum_{a\in A}v_{a,\tau }t_{a,\tau }$$ (1)

where *v*_*a*,*τ*_ represents the flow traveling via link *a* in period *τ*, which is obtained by solving the lower-level formulation; *t*_*a*,*τ*_ is the travel time on link *a* in period *τ*, which depends on *v*_*a*,*τ*_; *H* is the set of restoration periods; and *A* is the set of links. **s** = **[***s*_*a*_**]**, **v** = [*v*_*a*,*τ*_], **y** = [*y*_*a*,*τ*_] and **η** = [*η*_*a*,*τ*_] are the decision variable vectors, *s*_α_ is the starting period to restore a disrupted link, *y*_*a*,*τ*_ and *η*_*a*,*τ*_ are binary variables defined as:

$$y_{a,\tau }=\left\{\begin{array}{cc} 1 & \text{if}\;\text{link}\;a\;\text{is}\;\text{under}\;\text{restoration}\;\text{during}\;\text{period}\;\tau \\ 0 & \text{otherwise} \end{array}\right.$$ (2)

$$\eta_{a,\tau }=\left\{\begin{array}{cc} 1 & \text{if}\;\text{link}\;a\;\text{is}\;\text{fully}\;\text{restoredin}\;\text{period}\;\tau \\ 0 & \text{otherwise} \end{array}\right.$$ (3)

The ending period *e*_*a*_ for restoring the disrupted link *a* is defined by

$$e_{a}=s_{a}+T_{a},\forall a\in R$$ (4)

$$s_{a},e_{a}\in H,\forall a\in R$$ (5)

where *T*_*a*_ is the exact recovery periods required for restoring link *a* and *R* is the set of disrupted links to be restored. Constraint (5) defines the feasibility conditions of the decision variables.

According to assumption A4, the restoration of link *a* is continuous and uninterrupted; thus, the restoration period *τ* should satisfy the following equations:

$$\tau -s_{a}\leq M\left(1-y_{a,\tau }\right),\forall a\in R,\tau \in H$$ (6)

$$e_{a}-\tau \geq M\left(y_{a,\tau }-1\right),\forall a\in R,\tau \in H$$ (7)

where *M* is a sufficiently large positive number. In addition, resource constraints are often encountered during the restoration process, which may impact the sequencing of restoration activities. Let $\bar{C}$ denote the maximum unit of available resources in each period; then, the resources consumed in each period must satisfy the following condition:

$$\sum_{a\in R}C_{a}y_{a,\tau }\leq \bar{C},\forall \tau \in H$$ (8)

where *C*_*a*_ is resources required for restoring link *a*. Constraint (8) indicates that the total resources used in each period do not exceed the maximum resources for each period.

Once a disrupted link is fully repaired, it is functional with its designed capacity. As mentioned in constraint (3), *η*_*a*,*τ*_ is a binary variable indicating whether a disrupted link is functional in period *τ*; then, the following constraint needs to be implemented:

$$\eta_{a,\tau }\geq \eta_{a,\tau -1},\forall a\in R,\tau \in H$$ (9)

*(2) Lower-level formulation*.

With the advance of the restoration plan, the capacity of the disrupted links changes, which affects the decision-making behavior of travelers. Thus, the lower-level problem aims to establish a model for travelers’ route choice behavior and capture travelers’ responses to changes in the transportation network topology and capacity. The lower-level model is accordingly formulated as the UE traffic assignment model, and the objective function minimizing the sum of the integrals of the link travel time functions can be expressed as:

$$\underset{v}{\text{min}}\text{Q}({\text{T}}_{l})=\sum_{a\in A}\int_{\text{0}}^{v_{a,\tau }}t_{a,\tau }\left(v\right)dv$$ (10)

Note that the equilibrium flows in Eq (10) are subject to the following flow conservation and nonnegativity constraints:

$$\sum_{k\in K_{\tau }^{w}}f_{k,\tau }^{w}=g_{\tau }^{w},\forall w\in W$$ (11)

$$f_{k,\tau }^{w}\geq 0,\forall w\in W,k\in K_{\tau }^{w}$$ (12)

where $f_{k,\tau }^{w}$ is the flow traveling on path *k* connecting OD pair *w* in period *τ*; *W* is the set of OD pairs; $K_{\tau }^{w}$ is the set of paths connecting OD pair *w* in period *τ*; and $g_{\tau }^{w}$ is the travel demand of OD pair *w* in period *τ*. In addition, the following definitional constraint is required to further define the relationship between the link flow and path flow.

$$v_{a,\tau }=\sum_{w\in W}\sum_{k\in K_{\tau }^{w}}f_{k,\tau }^{w}\delta_{a,k,\tau }^{w},\forall a\in A$$ (13)

where $\delta_{a,k,\tau }^{w}$ is a binary variable defined as:

$$\delta_{a,k,\tau }^{w}=\left\{\begin{array}{cc} 1 & \text{if}\;\text{path}\;k\;\text{traverse}\;\text{link}\;a\;\text{in}\;\text{period}\;\tau \\ 0 & \text{otherwise} \end{array}\right.$$ (14)

#### 2.2.2 Stochastic bilevel programming

In practical situations, disruptive events often occur without warning, and it is impossible for people to have all the relevant information. Accordingly, the recovery time for each disrupted link is often stochastic, which poses a significant challenge to restoration activities. Using CVaR-R as a risk measure, we represent the uncertainty regarding the parameter *T*_*a*_ through a set of recovery time scenarios.

*(1) CVaR-R*.

As mentioned, the deterministic bilevel model uses the total travel time to measure the robustness of the restoration plan. However, decision-makers ignore the existence of worst-case scenarios under uncertainty, making the total travel time much greater than expected. In other words, a restoration plan under the stochastic case may not be the optimal for scenario *l*, leading to a deviation between the total travel time obtained via the plan and the ideal travel time [31, 32]. Consequently, for a given restoration plan, the regret of the total travel time *r*_*l*_(*u*) can be formulated as:

$$r_{l}\left(u\right)=m_{l}\left(u\right)-m_{l}^{*},\forall l\in L$$ (15)

where *m*_*l*_(*u*) is the total travel time associated with scenario *l* given by solution *u*; $m_{l}^{*}$ is the minimum total travel time associated with scenario *l*; and *L* is the set of recovery time scenarios. Moreover, each scenario has a certain probability of occurrence, and the corresponding probability *P*_*l*_ satisfies

$$\sum_{l=1}^{\left|L\right|}P_{l}=1,\forall l\in L$$ (16)

where |*L*| is the number of scenarios. CVaR-R at confidence level *α* is the mean regret of worst-case scenarios in which the collective probability of occurrence exceeds 1 **–***α*. Obviously, the regret is a discrete random variable that is particularly associated with scenarios, so we need to split the probability to ensure that the collective probability of worst-case scenarios is exactly 1 **–***α*. Let us sort the regret of the total travel time according to the corresponding value of *r*_*l*_(*u*), obtaining the order *r*_1_(*u*) < *r*_2_(*u*) <…< *r*_|*L*|_(*u*). To find the worst-case scenarios with collective occurrence probability 1 **–***α*, the unique index *π*_*α*_ should satisfy the following equation:

$$\sum_{l=1}^{\pi_{\alpha }}P_{l}\geq \alpha >\sum_{l=1}^{\pi_{\alpha }-1}P_{l}$$ (17)

The maximum regret $r_{\pi_{\alpha }}$ of the worst-case scenarios with collective occurrence probability 1 **–***α* is obtained via Eq (17). Consequently, the CVaR-R at confidence level *α*, i.e., *CVaR-R*_*α*_ (*u*), is given by

$$CVaR-R_{\alpha }(u)=\frac{1}{1-\alpha }\left[\left(\sum_{l=1}^{\pi_{\alpha }}P_{l}-\alpha \right)r_{\pi_{\alpha }}\left(u\right)+\sum_{l=\pi_{\alpha }+1}^{\left|L\right|}P_{l}r_{l}\left(u\right)\right]$$ (18)

*(2) Risk-based restoration optimization*.

As discussed in section 2.2.1, Eqs (1)–(14) determine the optimal restoration sequence of disrupted links for the deterministic case but not for the stochastic case. Nonetheless, these equations can then be used to calculate the minimum total travel time $m_{l}^{*}$.

In this subsection, we propose a risk-based stochastic model with a discrete distributed that minimizes the CVaR-R at confidence level *α* while considering the risk of worst-case scenarios with a cumulative probability of 1 –α. Thus, the upper-level objective function that is determined through the identification of worst-case scenarios to achieve the CVaR-R minimization is defined as follows:

$$\underset{s,v,y,\eta }{\text{min}}Z_{\alpha }=\frac{1}{1-\alpha }\left[\left(\sum_{l=1}^{\pi_{\alpha }}P_{l}-\alpha \right)r_{\pi_{\alpha }}\left(u\right)+\sum_{l=\pi_{\alpha }+1}^{\left|L\right|}P_{l}r_{l}\left(u\right)\right]$$ (19)

The next step is to transform the CVaR-R minimization problem for our upper-level model via the same formulation as Rockafellar and Uryasev [33]. Hence, Eq (19) can be equivalently written as follows:

$$\underset{s,v,y,\eta ,\zeta_{a}}{\text{min}}Z_{\alpha }=\zeta_{\alpha }\text{+}\frac{1}{1-\alpha }\left[\sum_{l=1}^{\left|L\right|}P_{l}\cdot \text{max}\left(r_{l}\left(u\right)-\zeta_{\alpha },0\right)\right]$$ (20)

subject to constraints (2)–(9) and (15)–(17), where *ξ*_*a*_ is a free decision variable defined as the quantile of *r*_*l*_(*u*) that exceeds the regret with probability *α*.

In the lower-level formulation, we have a UE problem shown in the objective function Eq (10) and constraints (11)–(14). In fact, the lower-level problem models the traffic assignment process for each period; that is, the uncertainty of the recovery time for each disrupted link has no impact on the formulation of the lower-level model. Subsequently, we obtain the lower-level model for the stochastic case:

$$\underset{v}{\text{min}}\text{Q}=\sum_{a\in A}\int_{\text{0}}^{v_{a,\tau }}t_{a,\tau }\left(v\right)dv$$ (21)

subject to constraints (11)–(14).

### 2.3 Network performance

We employ the unified network performance measure (UNPM) [34] to quantify the network performance. To facilitate the comparison of different settings, it is necessary to standardize the network performance measurements. Then, the UNPM and the network performance *φ*(*τ*) can be measured as

$$\varepsilon_{\tau }\left(v\right)=\frac{1}{\left|W\right|}\sum_{w\in W}\frac{g_{\tau }^{w}}{\lambda_{\tau }^{w}\left(v\right)},\forall \tau \in H$$ (22)

$$\varphi \left(\tau \right)=\frac{\varepsilon_{\tau }\left(v\right)}{\varepsilon_{\tau_{0}}\left(v\right)}\times 100\%,\forall \tau \in H$$ (23)

where *ε*_*τ*_(**v**) denotes the postdisaster UNPM in period *τ*; $\varepsilon_{\tau_{0}}\left(\text{v}\right)$ is the UNPM under the normal case; $\lambda_{\tau }^{w}\left(\text{v}\right)$ is the equilibrium travel time between OD pair *w* in period *τ*, which is obtained by solving the lower-level problem; and |*W*| is the number of OD pairs.

### 2.4 Solution method

#### 2.4.1 Scenario generation and reduction

A vital issue for a stochastic optimization model is how to quantify uncertainty. Among existing studies on the restoration scheduling problem, Monte Carlo simulation is the most common method for quantifying uncertainty because it allows probabilistic models to be associated with the proposed restoration problem [22, 24]. Monte Carlo simulation enables an increase in the number of simulations to reduce the error; however, in so doing, it also increases the difficulty of calculations. Thus, we apply an alternative technique, Latin hypercube sampling, to represent the uncertainty in recovery time for each damaged road segment. In particular, the Latin hypercube sampling technique is able to achieve high sampling accuracy for smaller sample sizes that is unmatched by Monte Carlo simulation.

Owing to the presence of uncertainty in the recovery time, there are diverse values of the stochastic parameter *T*_*a*_. The maximum and minimum recovery times for each disrupted link, namely, $T_{a}^{\text{max}}$ and $T_{a}^{\text{min}}$, are determined by the experience of decision-makers and the operation effectiveness of the engineering teams. In other words, $T_{a}^{\text{max}}$ and $T_{a}^{\text{min}}$ are typically considered upper and lower bounds on the expected recovery time for link *a*. To improve tractability, we conduct Latin hypercube sampling to generate a set of recovery time scenarios *L*, assuming that *T*_*a*_ is a uniformly distributed random integer in the range $\left[T_{a}^{\text{min}},T_{a}^{\text{max}}\right]$. By applying the Latin hypercube sampling technique, values in the range of 0 to 1 are divided into |*L*| disjoint intervals, and then |*L*| samples are selected to form a vector. We repeat the selection process for each disrupted link to form an |*L*|×|*R*| matrix. Then, when we multiply the matrix by $\left(T_{a}^{\text{max}}-T_{a}^{\text{min}}+1\right)$ and round up all the values in the matrix, |*L*| scenarios are constructed, and each scenario is characterized by equal probability $\frac{1}{\left|L\right|}$. Notably, a high number of initial scenarios can lead to better modeling of uncertainty as well as a higher computational burden. Consequently, a scenario reduction method should be implemented to obtain a small but representative set of recovery time scenarios. In this paper, the k-means++ [35] is employed to approximate the initial scenario set.

#### 2.4.2 Solution algorithm for bilevel model

The proposed bilevel optimization model is generally considered an NP-hard problem [36], which makes finding an exact solution more challenging. However, the brute force method can obtain the exact solution of the restoration scheduling problem, with a high computational cost, to traverse the entire solution space. Therefore, a novel GA is developed for solving the bilevel programming model. Previous research has shown that the GA can produce high-quality results for restoration scheduling problems [1, 24, 37]. The pseudocode for the proposed GA for the restoration scheduling problem is illustrated in Table 2.

**Table 2:** Pseudocode for the GA.

**Algorithm** GA
**Input:** maximum iterations *G*_max_, population size *N*, initial crossover rate *p*_*c*_, initial mutation rate *p*_*m*_
**Output:** minimum *CVaR-R*_*α*_ (*u*), optimal restoration sequence for disrupted links
**Step 1 for** each *l* **∈** *L*:
Solve the deterministic bilevel model to obtain the ideal total travel time $m_{l}^{*}$
**end for**
*G*←0
**Step 2** *N*←0
**for** *l* = 1 to |*L*| **do**
Compute the regret of the total travel time *r*_*l*_(*u*)
**End for**
**Sort** *r*_*l*_(*u*) **in an ascending order**
*N*←*N*+1
Calculate the fitness value of each solution *CVaR-R*_*α*_ (*u*)
**Step 3 if** *G* = *G*_max_ **then**
Return the final output
**else**
(a) Selection, crossover and mutation operations on the population
(b) *G*←*G*+1 and go to Step 2
**end if**

Note that $m_{l}^{*}$ and the fitness value are obtained by calculating the upper-level objective function for the deterministic and stochastic conditions, respectively, which involves solving the lower-level traffic assignment problem. For the lower-level problem, the model can be solved via the Frank‒Wolfe algorithm [38].

*(1) Solution representation*.

**Fig 2:**
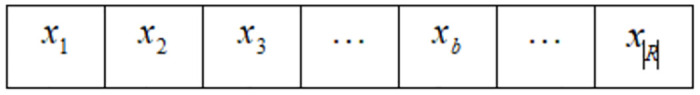
Solution representation.

When solving the proposed bilevel programming model, the GA is called twice, once to obtain $m_{l}^{*}$ if the recovery time is deterministic, and again to compute the fitness value under uncertainty. For simplicity, we apply the same solution representation and parameters for both calls. To apply the GA to solve our problem efficiently, the design of the solution representation should conform to the solution structure. Each solution representing the road segment restoration sequence consists of |*R*| elements, as shown in Fig 2. The *x*_*b*_ on the solution represents the index of the disrupted link. Notably, the value of *b* is an integer between 1 and |*R*|, and each number appears only once in each solution to indicate the continuity of each restoration activity.

*(2) Operators in GA*.

**Fig 3:**
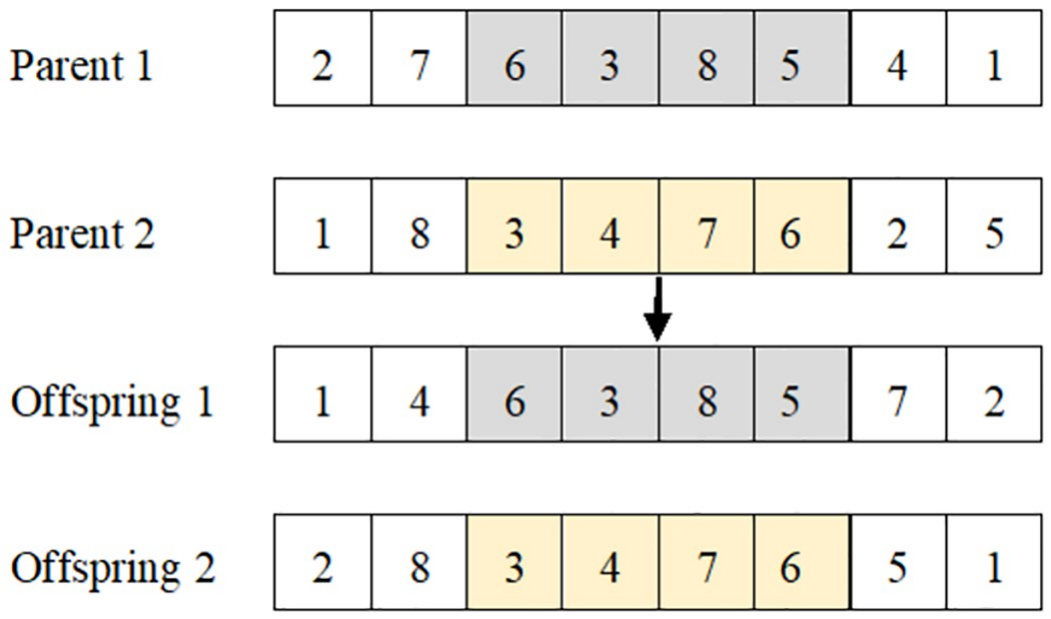
Crossover operation.

**Fig 4:**
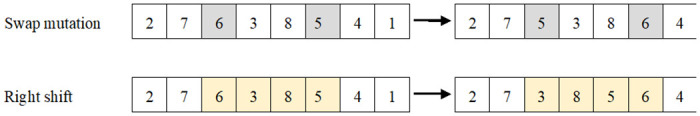
Mutation operation.

Three operators are designed to generate new solutions during the iteration process. A detailed description of the three operators is provided below:

1. Selection: According to the different ways of selecting individuals, the selection operators are divided into two categories: tournament selection and elite selection. The former is used to select two solutions as parent individuals, while the latter identifies the elite individual in the current population and then inserts it directly into the next generation.
2. Crossover: The crossover operator, which combines elements from two random high-quality parent individuals with a fixed probability, is employed to create new restoration sequences. Commonly used crossover operators applicable to serial numbering include partially mapped crossover operator and order crossover operator; however, partially mapped crossover requires gene conflict detection to maintain the feasibility of the solution. Thus, order crossover is applied, and the elements between two randomly chosen points are copied directly from parent individuals to offspring individuals. The applied crossover is illustrated in Fig 3.
3. Mutation: The mutation operator, which mutates the elements of the offspring produced via crossover with a specified probability, is developed to prevent the algorithm from falling into a local optimal solution. Two mutation strategies, swap and random right shift, are devised to increase population diversity. In the mutation stage, one of the two mutation operators is randomly selected to generate a feasible solution. The applied mutation operators are illustrated in Fig 4.

## 3 Numerical studies

In this section, we first describe two numerical case datasets and the relevant parameters. Numerical and sensitivity experiments are subsequently performed to analyze the properties of the proposed methodology.

### 3.1 Data description

In this study, two numerical cases are considered, which differ in terms of their topology and the link performance functions in the network:

*(1) Case 1: six-node network*.

**Fig 5:**
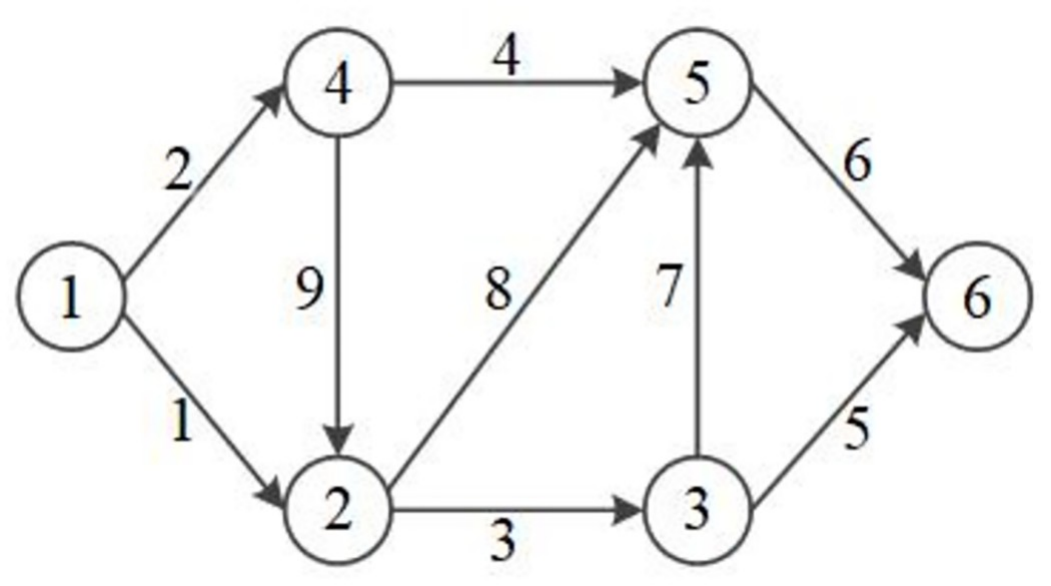
Six-node network.

We construct a six-node network, as represented in Fig 5, which consists of 6 nodes and 9 links. To complete the development of this network, an OD pair 1–6 with a travel demand set to 6.0 and the maximum units of available resources in each period set to 2 are considered. Furthermore, the link performance functions are given by *t*_1_(*v*_1_) = 8*v*_1_, *t*_2_(*v*_2_) = 25+0.5*v*_2_, *t*_3_(*v*_3_) = 2*v*_3_, *t*_4_(*v*_4_) = 25+0.5*v*_4_, *t*_5_(*v*_5_) = 50+*v*_5_, *t*_6_(*v*_6_) = 10*v*_6_, *t*_7_(*v*_7_) = 10+*v*_7_, *t*_8_(*v*_8_) = 5*v*_8_, and *t*_9_(*v*_9_) = 2+*v*_9_. Specifically, five links, namely, 4, 6, 7, 8, and 9, are disrupted.

*(2) Case 2: Nguyue—Dupuis network*.

**Fig 6:**
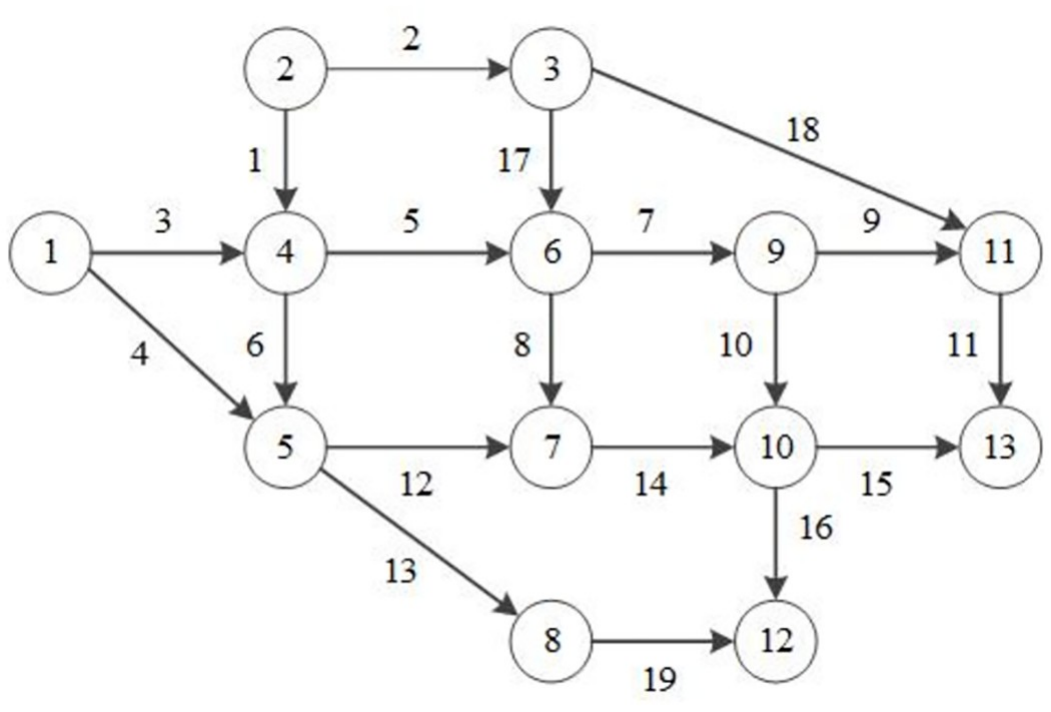
Nguyen–dupuis network.

We adopt the Nguyue—Dupuis network to test the algorithmic performance and illustrate the applications of the proposed model, as shown in Fig 6. In this example network, there are four OD pairs. The default travel demands associated with OD pairs 1–12, 1–13, 2–12 and 2–13 are 100, 300, 400 and 200, respectively. Details of the link attributes without disruption are referenced in a previous study [39]. Moreover, $t_{a,\tau }^{0}$ and *Q*_*a*,*τ*_ denote the free-flow travel time and traffic capacity on link *a* in period *τ*, respectively; then, the travel time is assumed to follow the Bureau of Public Roads (BPR) link performance function:

$$t_{a,\tau }=t_{a,\tau }^{0}\left[1+0.15{\left(\frac{v_{a,\tau }}{Q_{a,\tau }}\right)}^{\text{4}}\right],\forall a\in A,\tau \in H$$ (24)

In this test, we randomly select eight disrupted links, i.e., 2, 4, 9, 11, 12, 13, 17 and 19. Without further specification, all of these links are completely damaged, and only two links can be reconstructed in each period.

For convenience, throughout the study, the recovery time for each disrupted component is assumed to be an integer between 1 and 5. In the following subsections, we apply the GA to solve the numerical cases and compare the results with those of the particle swarm optimization algorithm (PSO) and the simulated annealing algorithm (SA). All three metaheuristic algorithms and the Frank‒Wolfe algorithm are coded via Microsoft Visual Studio Community 2022. The parameters considered in the metaheuristic algorithms are presented in Table 3, and the number of iterations for the Frank‒Wolfe algorithm is set to 500.

**Table 3:** Parameter settings in the metaheuristic algorithms.

GA parameters	Value	PSO parameters	Value	SA parameters	Value
Maximum iterations	200	Maximum iterations	200	Maximum iterations	200
Population size	20	Population size	20	Markov chain length	200
Initial crossover rate	0.9	Initial inertia weight	0.9	Initial temperature	100
Initial mutation rate	0.01	Acceleration constants	2	Temperature decay coefficient	0.95
		Largest velocity	30	Boltzmann constant	1

### 3.2 Results

#### 3.2.1 Comparison with the deterministic strategy

Based on the Latin hypercube sampling and scenario reduction method, 10 recovery time scenarios are formed.

The information about the disrupted links is listed in Table 4. It’s worth noting that each disrupted link is fully damaged, i.e., the capacity of those links drops to zero immediately after a given disruptive event.

**Table 4:** Information about disrupted links.

Link no.	Recovery time in each scenario									
	S1	S2	S3	S4	S5	S6	S7	S8	S9	S10
4	2	4	2	3	1	5	3	4	5	1
6	5	2	3	3	4	1	1	2	5	4
7	5	3	3	4	1	5	2	1	4	2
8	1	5	2	3	4	4	3	5	1	2
9	1	3	4	1	4	3	5	5	2	2

To verify the properties of the proposed approach for determining the restoration sequence of the disrupted links, we conduct experiments to compare the computational results obtained with the risk-based approach with those of two simulative empirical strategies. The first empirical strategy, namely, the ranking-based strategy, is based upon the idea that the increment in the total travel time when a link is removed from the network is regarded as a measure of link importance [34], which determines the order of the restoration activities. Another empirical strategy is to develop a restoration plan via the traffic flow in the undisrupted network and hence is also known as the flow-based strategy [20]. The results of each disrupted link are presented in Table 5.

**Table 5:** Results of disrupted links.

Link no.	Total travel time after removing the link	Traffic flow in the undisrupted network
4	526.1	0
6	589.9	4.1
7	521.9	0.9
8	546.3	3.2
9	558.3	2.2

For illustration purposes, the confidence level used for the risk-based stochastic optimization model is set at

*α* = 80%. Then, a brute force method was applied to enumerate all possible restoration sequences for the stochastic case to obtain the optimal CVaR-R value. The restoration sequences and the corresponding CVaR-R values under the three strategies are provided in Table 6. The boldface indicates that the two disrupted links begin to be restored at the same time. It can be seen that the risk-based strategy is more efficient than the other two restoration strategies are, with CVaR-R values reduced by more than 31% and 38%, respectively.

**Table 6:** Restoration results for the three strategies.

Strategy	Restoration sequence	Optimal CVaR-R value
Ranking-based	**6**-**9**-8-4-7	901.5
Flow-based	**6**-**8**-9-7-4	950.5
Risk-based	**8**-**9**-6-4-7	686.8

**Fig 7:**
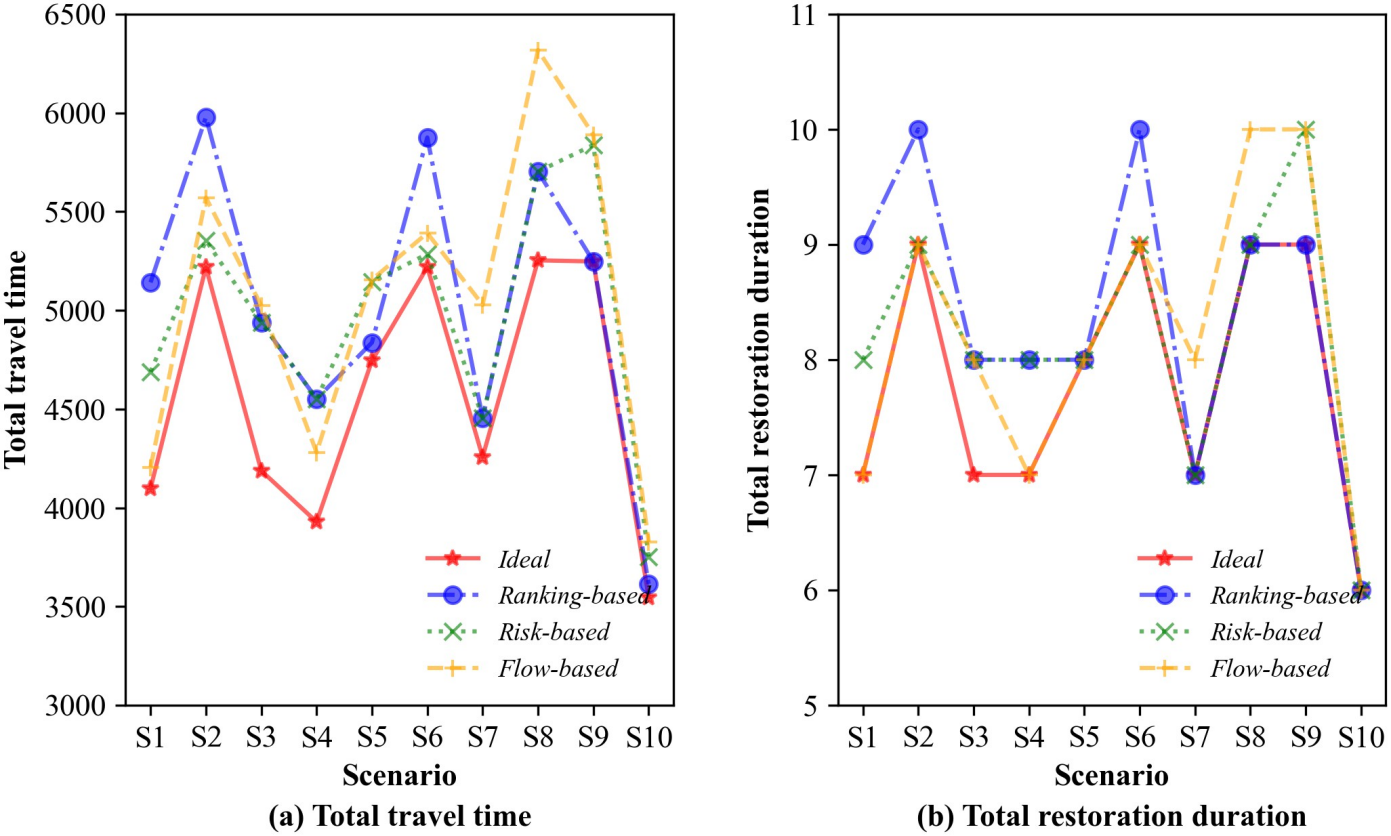
Comparison of total travel time and total restoration duration.

For further comparison, we also plot the changes in the total travel time curve and total restoration duration curve for the different restoration strategies in Fig 7. Fig 7(a) shows a large gap between the total travel time of the ranking-based strategy, the flow-based strategy and the ideal strategy (i.e., the restoration plan obtained under the deterministic case), which can lead to relatively large regret values for most scenarios. By comparison, the risk-based strategy obtains a smaller value of the regret of the total travel time. In addition, more than seven lower values of total travel time obtained by the risk-based strategy out of 10 scenarios imply that the strategy allows a higher quality of restoration to be achieved. As shown in Fig 7(b), the total restoration duration for scenario S9 under the ranking-based strategy is 9, while it is 10 under the risk-based strategy. Furthermore, in the other 9 scenarios, the risk-based strategy obtains shorter total restoration durations. Compared with the flow-based strategy, the risk-based strategy obtains eight lower values of total restoration durations. It is obvious that the risk-based strategy results in a shorter total restoration duration for the identified 10 scenarios, suggesting that the risk-based strategy can accomplish restoration activities more quickly than the other two restoration strategies can. These results indicate that a significant benefit in adopting the restoration sequence obtained with the risk-based strategy over that obtained with the ranking- or flow-based strategy for improving system resilience and reducing system risk under uncertainty.

#### 3.2.2 Algorithm performance comparison

This subsection provides numerical examples to evaluate the performance of the newly proposed GA and to compare it with the brute force method, PSO and SA. 10 independent recovery time scenarios are generated via the scenario generation and reduction methods, as shown in Table 7. The brute force method is applied to enumerate all the possible solutions in each benchmark to obtain the optimal restoration sequence. For the sake of fairness, 20 runs for the metaheuristic algorithms are performed using the same random seeds. Notably, the link flow determined by the lower-level model affects the optimal solution of the upper-level model. Therefore, we vary the convergence tolerance from 1.0e-2 to 1.0e-5 for the traffic assignment problem. The results showed that the restoration sequence, i.e., **2**-**11**-12-4-13-9-17-19, remains unchanged at all convergence tolerances. The boldface indicates that the two disrupted links begin to be restored at the same time. Consequently, the convergence tolerance for all experiments in the following is set to 1.0e-2.

**Table 7:** Information about disrupted links of the Nguyue–Dupuis network.

Link no.	Recovery time in each scenario									
	S1	S2	S3	S4	S5	S6	S7	S8	S9	S10
2	4	5	1	5	3	1	4	2	2	3
4	1	2	1	5	2	4	5	3	4	3
9	1	3	5	5	2	2	4	4	3	1
11	5	1	2	2	3	4	3	1	5	4
12	3	1	4	5	5	4	3	2	1	2
13	2	1	5	3	2	3	4	4	1	5
17	4	5	3	3	1	2	4	2	5	1
19	5	3	3	5	1	2	2	4	4	1

A summary of the numerical results obtained via the different methods is provided in Table 8. It is worth pointing out that the GA can indeed be applied to the restoration scheduling problem under uncertainty. Moreover, its advantages are reflected mainly in the following aspects: first, the GA obtains the same fitness as the brute force method does, and its success rates of obtaining the best fitness are 90% and 25% higher than those of PSO and SA, respectively. Second, in the premise of ensuring high-quality solutions, the GA consumes much less computation time than SA does.

**Table 8:** Comparison between the GA and the other methods.

	Brute force	PSO	SA	GA
Average of fitness	104957.7	114820.2	106344.2	104957.7
Average CPU time	35662	744.2	4321.5	675.3
Best fitness	104957.7	104957.7	104957.7	104957.7
Number of best solutions obtained	20	2	15	20

**Fig 8:**
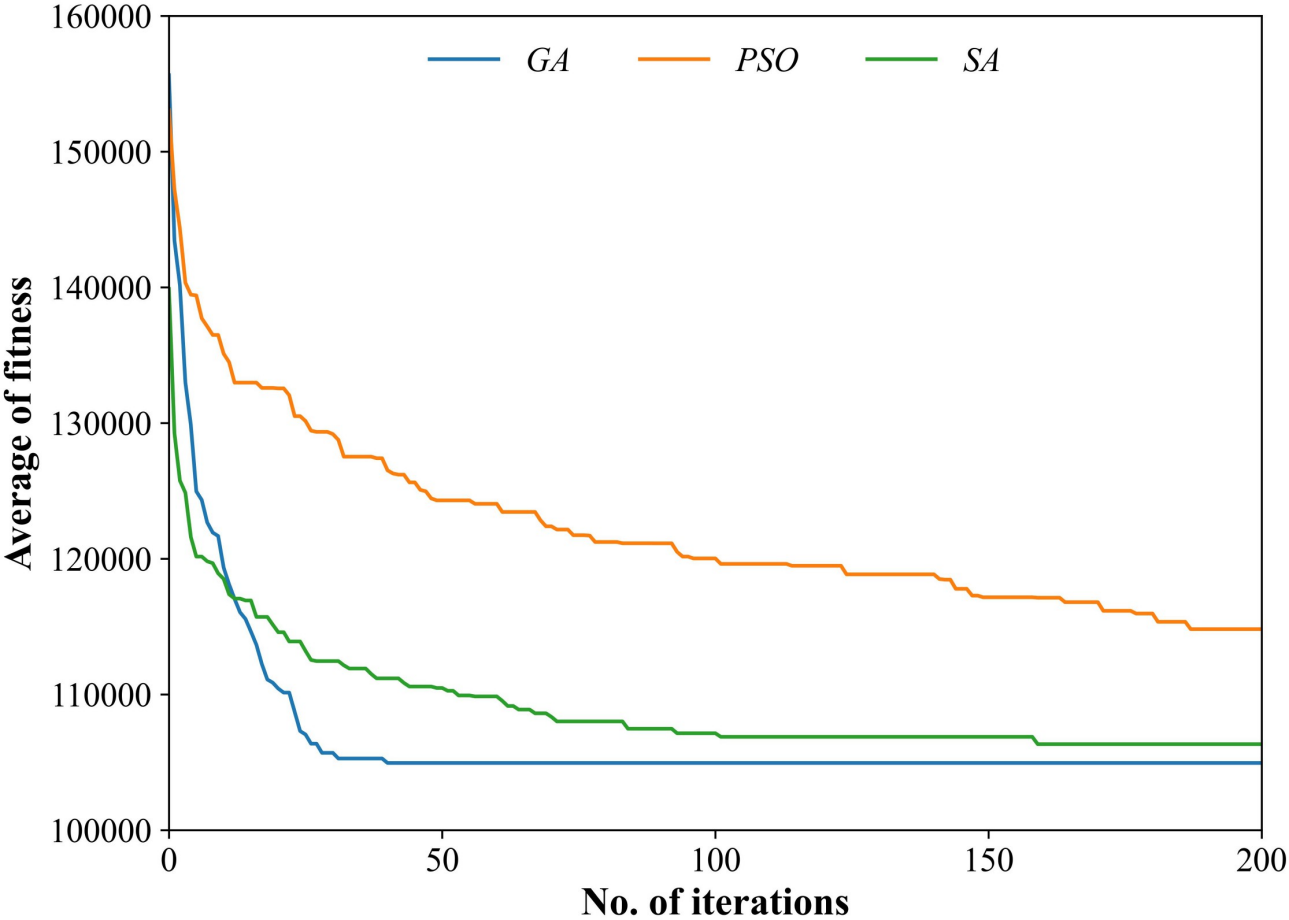
Performance of the three algorithms.

Fig 8 depicts the performance of the GA, PSO and SA over generations. Although these three algorithms use the same solution representation and termination criterion, they lead to different convergence trajectories. As shown in Fig 8, the proposed GA converges at the 41st generation, which indicates that, compared with PSO and SA, the GA has a fast convergence speed and good stability.

#### 3.2.3 Sensitivity analysis

In general, for a given transportation network, three main factors affect the progress of system risk recession and system resilience improvement: confidence level, travel demand and available resources. Owing to the uncertainty associated with disruptive events and restoration activities, sensitivity analysis regarding these factors is worth investigating.

*(1) Effect of confidence level*.

To investigate the effect of the confidence level on the solution, we change *α* from 0% to 90% with an increment of 10%. The restoration sequences with different confidence levels can be seen in Table 9. The bold numbers indicate meanings that are consistent with those in section 3.2.1. The optimization results in Table 9 show that the restoration sequence is not always identical at each confidence level, and the links that first start to be restored are different with various values of *α*, while link 4 is always ranked in fourth place. Notably, link 17 is the last link to be restored, which means that its importance is relatively weaker than that of other links at medium low values of *α*. Therefore, it can be found that the confidence level affects the rankings of the restoration activities in the transportation network, which may be significant or minor. Our model solutions provide guidance for optimizing the restoration sequence of disrupted infrastructures to deliver services more effectively for confidence-sensitive types during the restoration process.

**Table 9:** Optimal restoration results under different confidence levels.

Confidence level	Restoration sequence
0%-30%	**11**-**12**-2-4-13-19-9-17
40%	**11**-**12**-2-4-9-13-19-17
50%-60%	**2**-**12**-11-4-9-13-19-17
70%-90%	**2**-**11**-12-4-13-9-17-19

**Fig 9:**
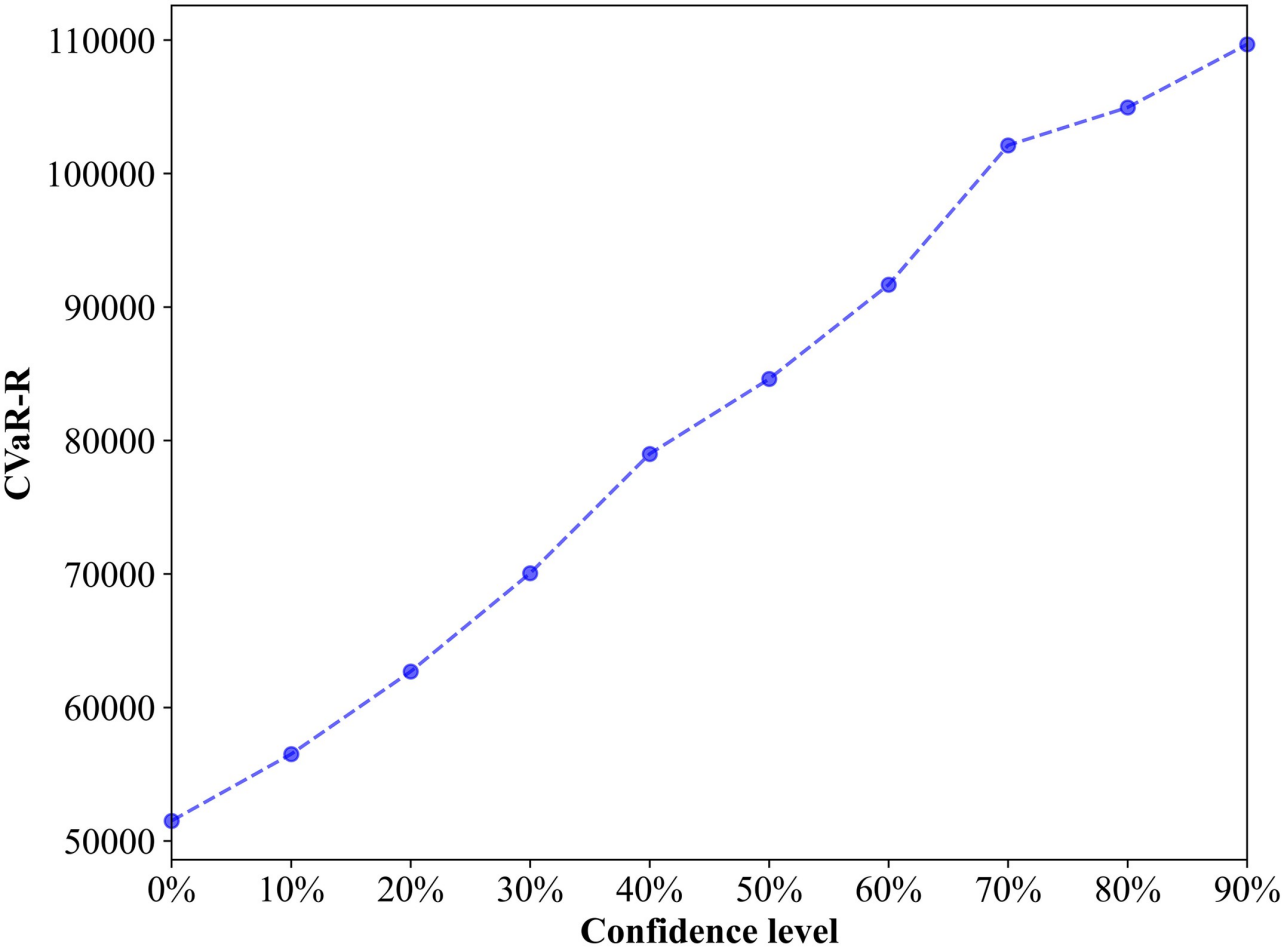
Impact of the confidence level on the CVaR-R value.

To facilitate the explanation, Fig 9 plots the changes in the value of CVaR-R with respect to the changes in the value of *α*. It is obvious that the CVaR-R value is higher at a larger value of *α*. This result is as expected, as a higher *α* induces a lower collective occurrence probability of worst-case scenarios, which is considered in the mean regret of worst-case scenarios. In other words, the higher the confidence level is, the greater the degree of risk aversion, and the higher the probability that the loss caused by the risk will not exceed this CVaR-R value. Moreover, as shown in Fig 9, when the overall value changes between 0% and 90%, the CVaR-R value increases sharply when 60% ≤ *α* ≤ 70%. This means that with increasing *α*, decision-makers have the greatest degree of mean regret for the total travel time. The above results confirm that the value of *α* plays a significant role in the decision-making of managers, suggesting that decision-makers pay limited attention to the worst-case scenarios because of less risk aversion when *α* is small.

**Fig 10:**
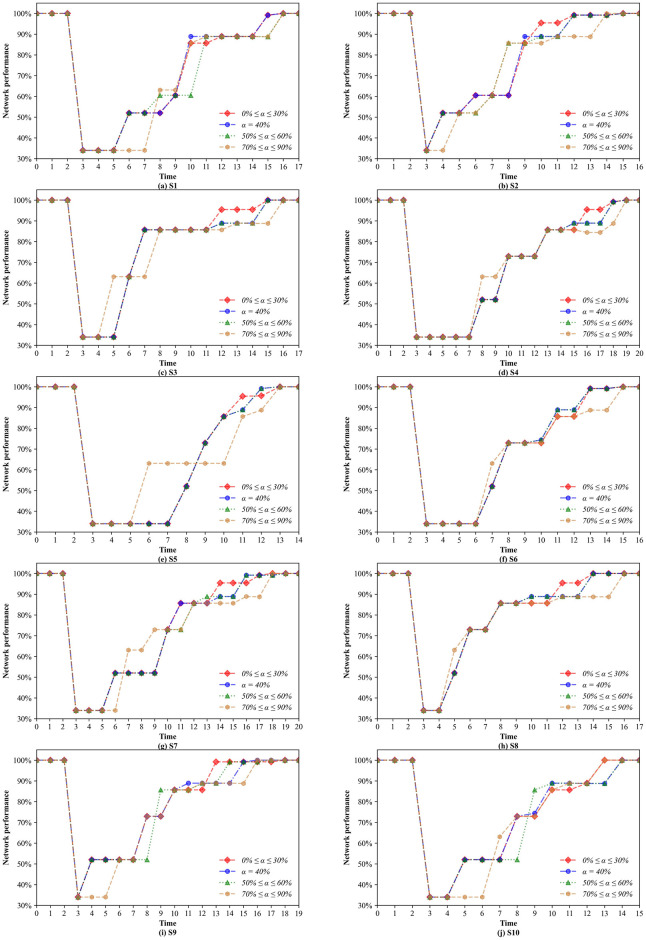
Changes in the network performance under different confidence levels.

Furthermore, we investigate how the network performance changes under each of the 10 scenarios for solutions with different confidence levels. To illustrate the restoration process clearly, we add two additional normal periods before and after the restoration periods, where the network performance is 100%. Fig 10 presents the network performance restoration trajectories of the 10 scenarios. Obviously, the network performance gradually returns to normal with the restoration of the disrupted infrastructures. On the other hand, it can be seen that the confidence level indeed has an effect on the network performance. The reason is that the changes in the value of *α* lead to the reformulation of the restoration sequence. Nonetheless, there is no significant negative correlation between the network performance and the confidence level. Finally, the main reason for the overlapping curves is that the worst-case scenarios identified under different confidence levels include the same scenarios.

*(2) Effect of demand level*.

This subsection elaborates the effect of the demand level on the solution (with *α* = 80%). In the following experiments, we introduce a demand scale parameter *ρ*, where the original demand matrix is multiplied by 0.5, 1.0, 1.5 and 2.0. The summary of the restoration results under the four demand levels is presented in Table 10; in this table, the middle column shows the changes in the restoration sequence under different demand levels, and the last column shows the corresponding CVaR-R values.

**Table 10:** Optimal restoration results under different demand levels.

Demand level	Restoration sequence	Optimal CVaR-R value
0.5	**4**-**12**-9-19-2-13-11-17	17120.6
1.0	**2**-**11**-12-4-13-9-17-19	104957.7
1.5	**11-12**-2-4-9-13-19-17	382505.8
2.0	**2**-**12**-17-4-11-19-13-9	2099192.6

According to the results in Table 10, one can find that the restoration sequence varies with the demand level. More interestingly, except when the demand level is 0.5, link 4 is always the 4th to be restored in the other three cases. The reason is that under low-level demand, the link capacity does not reach the saturation point, and the traffic congestion does not appear in the transportation network when it is necessary to ensure connectivity for all OD pairs. In addition, we also note that OD pairs 2–13 must be restored first regardless of the demand level; that is, ensuring that at least one of links 11 and 12 is functional is important. Table 10 also presents the CVaR-R value with various demand levels. As expected, the CVaR-R grows with increasing demand, but the extent of change in the values varies. This finding demonstrates that the demand level indeed has an effect on the CVaR-R value, although the regret is less intense at low demand. On the other hand, it shows that the value of risk-based optimization modeling becomes increasingly important when we are interested in outcomes with high demand levels and high risk averse.

**Fig 11:**
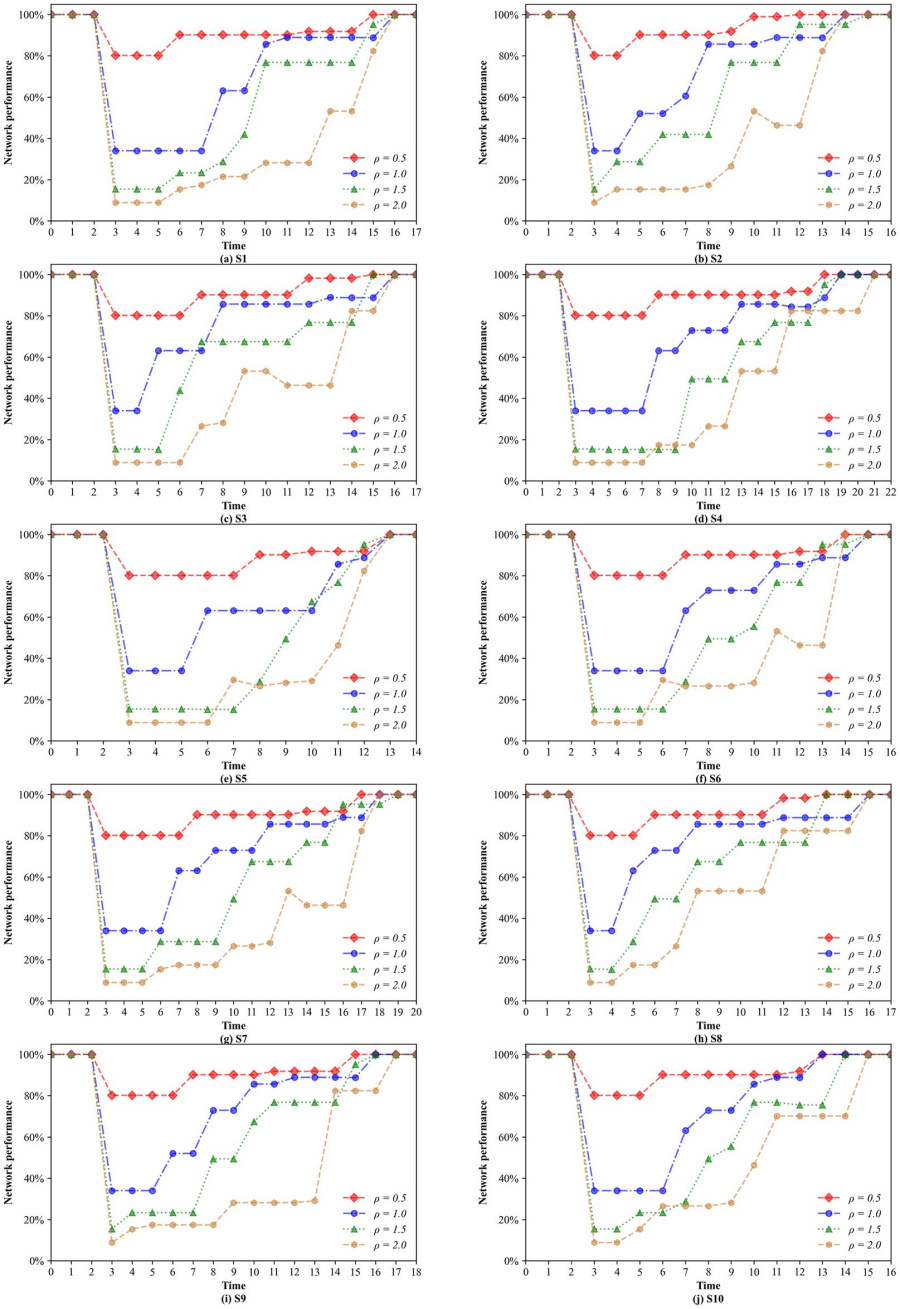
Changes in the network performance under different demand levels.

Fig 11 shows the effects of different demand levels on the network performance. It is obvious that after the occurrence of disruptive events, the larger the value of *ρ* is, the greater the decrease in network performance under uncertainty is. However, in the middle and latter periods of the restoration process, high demand does not necessarily mean low network performance. Interestingly, there are inflection points in the performance curves of scenarios S2, S3, S6 and S7 when *ρ* = 2.0. This is an anomaly implying that the newly restored links play a negative role in the next short period of time. This phenomenon could be considered a Braess paradox because adding new links to the transportation network degrades the network performance instead, which contrasts with our intuition. The above results demonstrate that once the demand reaches a certain level, the paradox may occur even if the recovery time for each disrupted link is uncertain. Moreover, compared with the network performance curve of other 9 scenarios, that of scenario S5 is relatively flat and has a shorter restoration duration, which leads to a higher regret value. This means that there is a high probability that scenario S5 is one of the worst-case scenarios even under different demand levels. It is verified that, except when *ρ* = 1.0, scenario S5 is indeed one of the worst-case scenarios under all demand levels.

*(3) Effect of resource level*.

In order to get the transportation network return to its normal state as soon as possible, how to rationalize the use of resources is the key to the entire restoration process. Obviously, the worst part is that only one link can be restored at a time, and the best part is that all the disrupted links can be restored at the same time. In what follows, we illustrate the restoration results under different resource levels. In the test, the maximum unit of available resources in each period varies from 1 to 8, with a predefined confidence level *α* = 80%.

The changes in the restoration sequences associated with the eight levels are presented in Table 11. Notably, the restoration sequences are consistent with expectations when the available resources are 1 and 8, which is in line with reality. Moreover, the table shows that link 11 is the preferred link in all the experiments except when the resource level is 1, while link 12 also becomes one of the first choices once the resource level reaches 3. From Table 11, it can be inferred that the restoration sequence changes with increasing the resource level; however, when the resource level is increased to a certain level (e.g., a threshold of 5 in this study), the sequence remains essentially unchanged. Indeed, a resource level of more than 6 can be considered unnecessary under uncertainty. That is, if there are sufficient resources to restore most of the infrastructures in each period, the entire restoration period is shortened, but the resources do not have a significant effect on the restoration sequence. The above results indicate that the proposed bilevel programming can effectively solve the restoration scheduling problem while considering resource constraints and risk aversion. Additionally, a conclusion can be drawn that there is a balance between the determination of the restoration sequence and the resource level.

**Table 11:** Optimal restoration results under different resource levels.

Resource level	Restoration sequence
1	12-4-11-2-13-19-9-17
2	**2**-**11**-12-4-13-9-17-19
3	**2**-**11**-**12**-4-9-13-19-17
4	**11**-**12**-**4**-**9**-2-13-19-17
5	**11**-**12**-**13**-**2**-**19**-4-17-9
6	**11**-**12**-**13**-**2**-**19**-**4**-9-17
7	**11**-**12**-**13**-**2**-**19**-**4**-**9**-17
8	**11**-**12**-**13**-**2**-**19**-**4**-**9**-**17**

**Fig 12:**
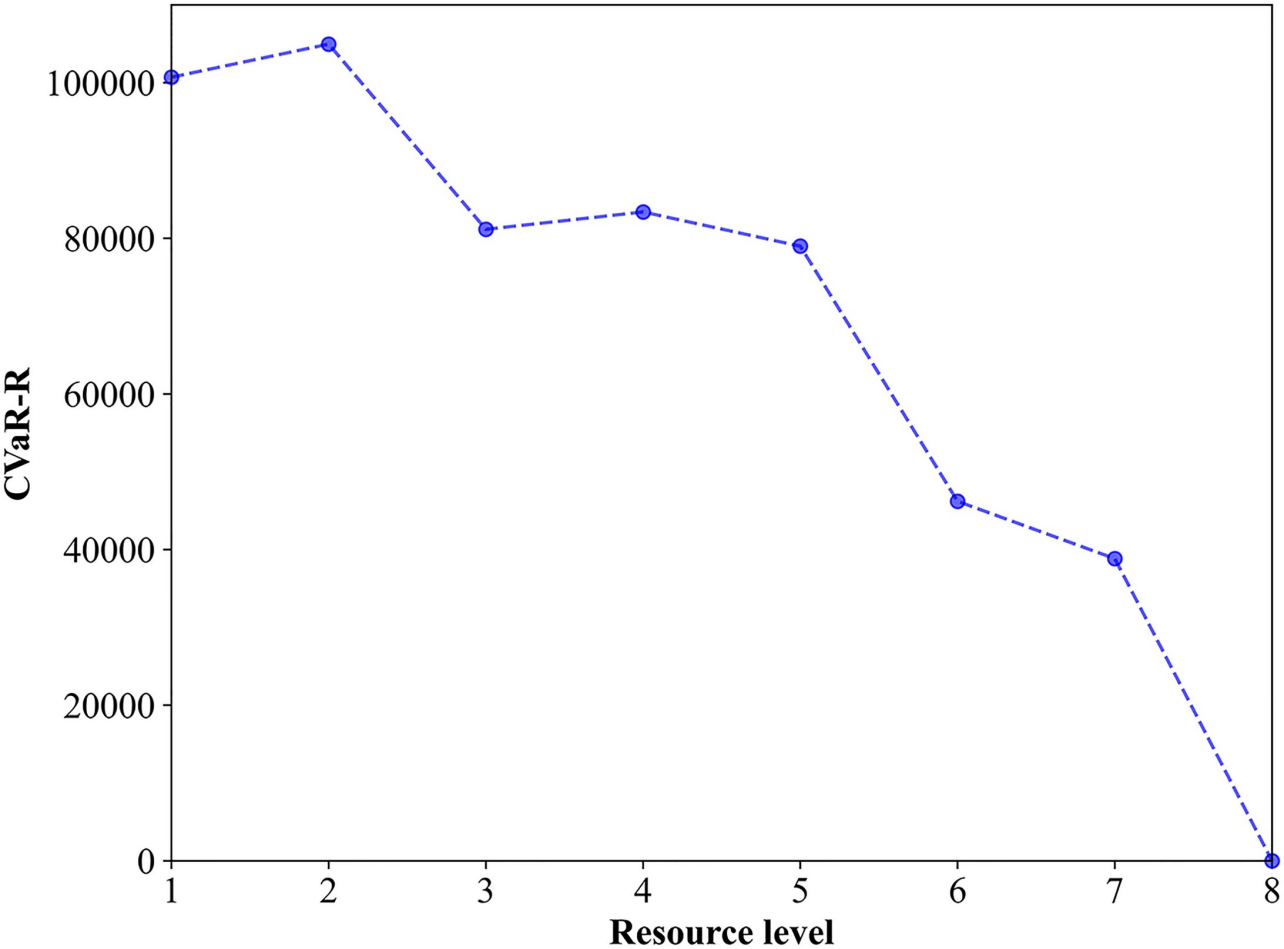
Impact of the resource level on the CVaR-R value.

To further analyze the effect of resources on the solution, Fig 12 shows how the CVaR-R values vary with different resource levels. Interestingly, the CVaR-R value reaches a maximum of 104957.7 when the resource level is 2. This result demonstrates that adding more resources does not reduce the CVaR-R value. This phenomenon can also be considered considered a paradox because it is contrary to our intuition that increasing the available resources would decrease the mean regret of worst-case scenarios. There could be two reasons for this phenomenon: (1) the basic access requirements for the transportation network are met and its traffic congestion is rapidly alleviated, as long as the resource level reaches 2 (as is the case for our example); and (2) as the resource level increases, *m*_*l*_(*u*) and $m_{l}^{*}$ get reduced for all scenarios; however, the difference value *r*_*l*_(*u*) does not necessarily decrease. For example, the number of scenarios where *r*_*l*_(*u*) instead increases is 8 when the level is 2, which is more than any other resource level. However, the overall CVaR-R value tends to decrease as the resource level increases. Therefore, in the case of insufficient available resources, it is most necessary to determine the quantity of available resources and perform risk-based optimization modeling to reduce the mean regret of worst-case scenarios.

**Fig 13:**
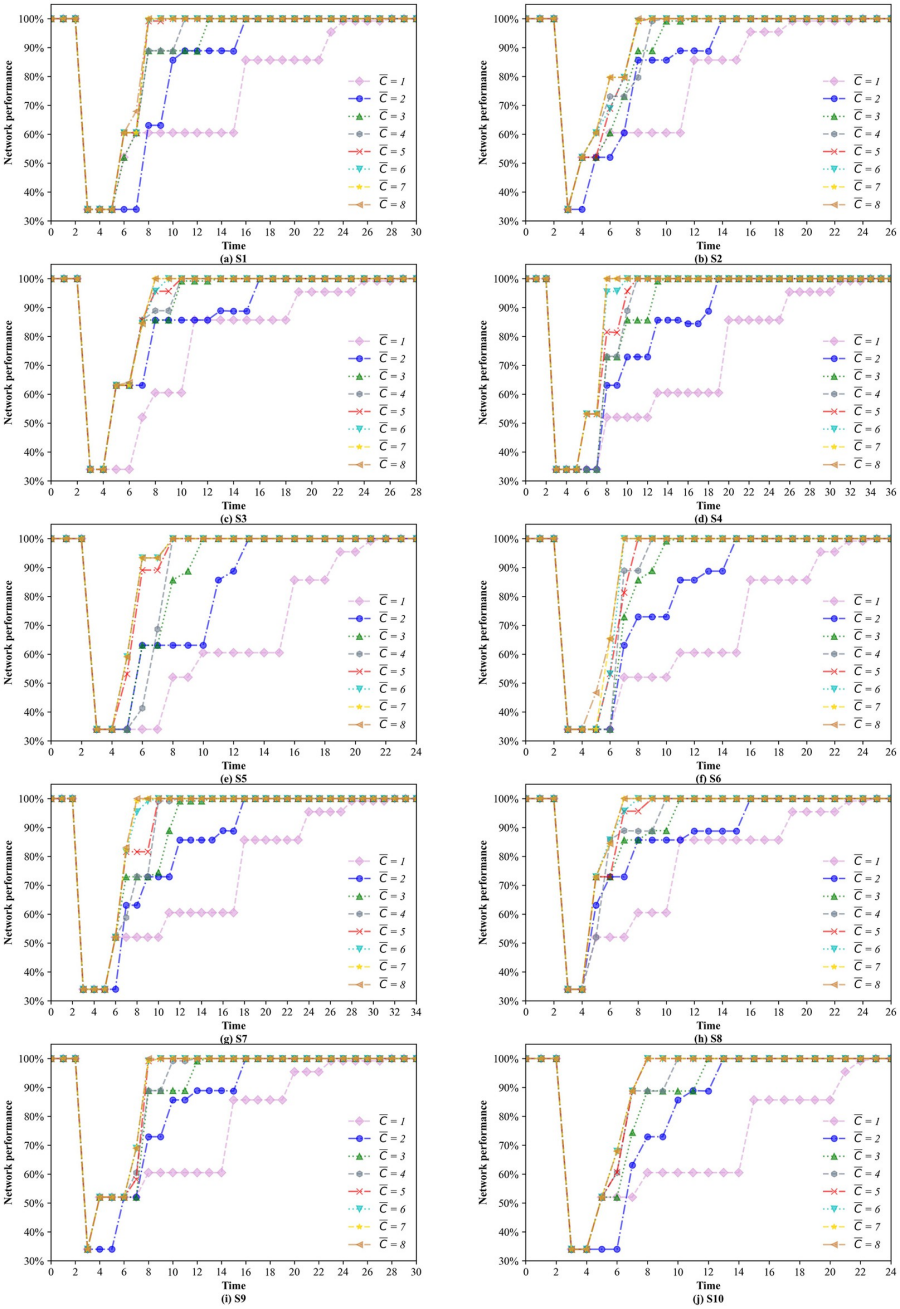
Changes in the network performance under different resource levels.

In addition, we also plotted the changes in network performance under different resource levels for the 10 scenarios (see Fig 13). Among the 10 uncertain scenarios, the highest network performance is achieved when the resource level is equal to 8, and it increases over time, this is mainly because more disrupted links can be restored at the same time, thus restoring the network performance to its normal state more quickly. The comparisons between the other resource levels illustrate that there is not necessarily a positive correlation between the quantity of available resources and the network performance.

A comparison of the various subplots in Fig 13 reveals that the network performance curves that consider the uncertainty of the recovery time are very different. For example, in scenario S1, the network performance curves are simpler, where the overlap is greater. In other words, scenario S1 is more likely to be one of the worst-case scenarios under different resource levels. Fig 13 demonstrates that the resource level affects network performance under uncertainty, although the effect may be significant or slight.

### 3.3 Discussion

In previous studies on transportation infrastructure restoration, the uncertainty associated with disruptive events has often not been considered. Most of the studies take resilience measures as the main evaluation indicators while ignoring the impact of decision-makers’ risk attitudes on the restoration plan. When the recovery time is unknown, ignoring the existence of worst-case scenarios under uncertainty may lead to greater deviations from the ideal plan. To address these issues, this study couples resilience and risk measures to develop a novel methodology, which makes the restoration plan more scientific.

In comparison with existing studies that consider the flow and ranking of links as transportation network restoration strategies, the results of this study ensure the minimization of total travel time and total restoration duration under the most uncertain scenarios. The numerical results also indicate that the proposed GA can find the exact solution with a success rate of 100% and an evidently reduced computation time. In addition, changes in the confidence level, travel demand and available resources under uncertainty not only affect the determination of the optimal restoration sequence but also significantly affect network performance. The Braess paradox may arise, especially when the traffic demand reaches a certain level (which means that the network performance is better than that after restoring a new link). Furthermore, a high resource level speeds up the process of restoring disrupted infrastructures but does not necessarily adjust the restoration sequence or reduce the mean of the risk incurred by the worst-case scenarios.

This study explores the application of risk- and resilience-based restoration optimization of transportation infrastructures under uncertainty. The proposed methodology is not limited to the rehabilitation of transportation networks; thus, it can be helpful in other contexts, such as power distribution network restoration projects and road network capacity improvement projects. However, one should note that our work also has several limitations when applied in practice. For example, we assume that the travel demand is fixed, but in reality, some travelers may not be able to tolerate long waits, which may lead to a reduction in demand, or they may choose multiple modes of transportation during their trip. In addition, our current analysis only involves a single resilience measure and does not consider a trade-off between multiple measures. Moreover, the results of the validation using numerical examples are one-sided because the recovery speed of each disrupted transportation infrastructure is different. Therefore, to further verify the credibility of the proposed methodology, real-world case study data are required.

## 4 Conclusions

Motivated by considering the uncertainty in the restoration scheduling problem, this study develops a bilevel programming model. The upper-level problem is to determine the optimal restoration sequence by minimizing the CVaR-R while considering resource constraints, whereas the lower-level problem is a traffic assignment problem that captures travelers’ response to changes in network topology and capacity. Therefore, in other words, the model aims to minimize the CVaR-R of the total travel time in the presence of recovery time uncertainty for the sake of enhancing the system resilience and reducing the system risk while considering travelers’ route choice behavior. The proposed model addresses two important challenges facing restoration scheduling, which are the uncertainty associated with the recovery time of restoration activities and risk aversion. For the former, the uncertainty of the recovery time is addressed by sampling from a predetermined probability distribution through a Latin hypercube technique. For the latter, risk aversion is achieved mainly by transforming the model from the deterministic case to the stochastic case. A highly efficient algorithm framework that integrates a novel genetic algorithm and the Frank—Wolfe algorithm is subsequently proposed to solve the stochastic optimization model. The numerical results demonstrate that the proposed methodology is a better method for solving the restoration scheduling problem than traditional restoration strategies and algorithms are.

Looking ahead, there are several directions in which this study can be extended. First, this study focuses mainly on a single road traffic mode, ignoring the interdependent effects between private, transit and metro modes. It would be interesting to extend the proposed model to a multimodal transportation system. Second, the model in the present study assumes that the travel demand between each OD pair is stationary during the recovery process. Indeed, relaxing this assumption by extending our model to capture the dynamics of travel demand over time would add more flexibility to the optimization model. Third, only one resilience measure, total travel time, is considered when quantifying resilience; however, there are many other resilience measures, such as resilience loss, total restoration duration and skewness of the restoration trajectory. Therefore, considering a bilevel multiobjective programming model under uncertainty and a risk-averse approach can also be investigated as a future research direction. Last but not least, this study focuses on verifying the effectiveness of the bilevel model through numerical examples and does not consider the application in the real world. However, other realistic influencing factors could be incorporated into the proposed model to form a more scientific optimization model. Therefore, numerical experiments based on real-world transportation networks are necessary in future research.

## Supporting information

S1 File (DOCX)
