# Supplementary material for: Risk and resilience-based restoration optimization of transportation infrastructures under uncertainty
Source: PLoS One. 2024 Aug 1;19(8):e0308138. doi: 10.1371/journal.pone.0308138 (PMC11293662; doi:10.1371/journal.pone.0308138)
Supplement: S1 File — (DOCX) [file pone.0308138.s001.docx]

Supporting information

**The source code for reproducing the experiments in this study are available in the 1st author’s GitHub repository:** [Jualin2021/RSP-Test (github.com)](https://github.com/Jualin2021/RSP-Test)**.**
